# *O*-(*tert*-Butyl­dimethyl­silyl)tris­(*O*-4-methyl­phenyl­sulfon­yl)penta­erythritol

**DOI:** 10.1107/S160053680802117X

**Published:** 2008-07-12

**Authors:** Shu-Xian Li, Hua-Min Li, Zhong-Lin Lu, Hoong-Kun Fun, Suchada Chantrapromma

**Affiliations:** a Department of Chemistry, Beijing Normal University, Beijing 100875, People’s Republic of China; b Department of Chemistry, Handan College, Handan, Hebei 056005, People’s Republic of China; c X-ray Crystallography Unit, School of Physics, Universiti Sains Malaysia, 11800 USM, Penang, Malaysia; d Crystal Materials Research Unit, Department of Chemistry, Faculty of Science, Prince of Songkla University, Hat-Yai, Songkhla 90112, Thailand

## Abstract

In the title compound [systematic name: (*tert*-butyl­dimethyl­silyl)methane­triyl tris­(4-methyl­benzene­sulfonate)], C_32_H_44_O_10_S_3_Si, the central C atom and the Si^IV^ center are in a tetra­hedral configuration. The inter­planar angles between pairs of the three benzene rings of the 4-methyl­phenyl­sulfonyl units are 41.15 (10), 18.11 (10) and 44.09 (10)°. C—H⋯π inter­actions are observed in the crystal structure. Mol­ecules are linked into screw chains along the *b* axis by weak C—H⋯O inter­actions. Weak intramolecular C—H⋯O hydrogen bonds are also present.

## Related literature

For bond-length data, see: Allen *et al.* (1987 ▶). For background and applications of radioimmuno imaging, radioimmuno therapy and hypoxia markers, see, for example: Abdel-Jalil *et al.* (2006 ▶); Monge *et al.* (2001 ▶); Nagasawa *et al.* (2006 ▶).
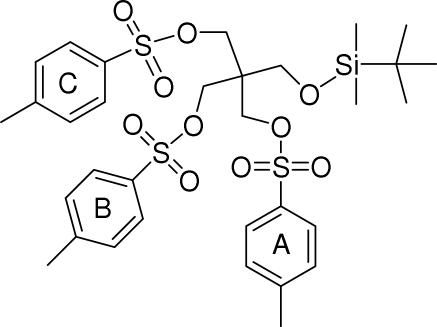


## Experimental

### 

#### Crystal data

C_32_H_44_O_10_S_3_Si
*M* *_r_* = 712.94
Monoclinic, 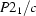

*a* = 19.0551 (3) Å
*b* = 16.4726 (3) Å
*c* = 11.6751 (2) Å
β = 100.425 (1)°
*V* = 3604.17 (11) Å^3^
*Z* = 4
Mo *K*α radiation
μ = 0.29 mm^−1^
*T* = 100.0 (1) K
0.51 × 0.22 × 0.21 mm

#### Data collection

Bruker SMART APEX2 CCD area-detector diffractometer
Absorption correction: multi-scan (*SADABS*; Bruker, 2005 ▶) *T*_min_ = 0.865, *T*_max_ = 0.941
45850 measured reflections
10507 independent reflections
7229 reflections with *I* > 2σ(*I*)
*R*_int_ = 0.061

#### Refinement

*R*[*F* ^2^ > 2σ(*F* ^2^)] = 0.049
*wR*(*F* ^2^) = 0.128
*S* = 1.08
10507 reflections
423 parameters
H-atom parameters constrained
Δρ_max_ = 0.57 e Å^−3^
Δρ_min_ = −0.37 e Å^−3^

### 

Data collection: *APEX2* (Bruker, 2005 ▶); cell refinement: *APEX2*; data reduction: *SAINT* (Bruker, 2005 ▶); program(s) used to solve structure: *SHELXTL* (Sheldrick, 2008 ▶); program(s) used to refine structure: *SHELXTL*; molecular graphics: *SHELXTL*; software used to prepare material for publication: *SHELXTL* and *PLATON* (Spek, 2003 ▶).

## Supplementary Material

Crystal structure: contains datablocks global, I. DOI: 10.1107/S160053680802117X/at2586sup1.cif

Structure factors: contains datablocks I. DOI: 10.1107/S160053680802117X/at2586Isup2.hkl

Additional supplementary materials:  crystallographic information; 3D view; checkCIF report

## Figures and Tables

**Table 1:** Hydrogen-bond geometry (Å, °)

*D*—H⋯*A*	*D*—H	H⋯*A*	*D*⋯*A*	*D*—H⋯*A*
C2—H2*A*⋯O5^i^	0.97	2.57	3.133 (2)	117
C2—H2*B*⋯O2	0.97	2.56	2.902 (2)	101
C4—H4*A*⋯O6^i^	0.93	2.54	3.446 (3)	166
C10—H10*B*⋯O1	0.97	2.50	2.856 (2)	102
C10—H10*B*⋯O6	0.97	2.54	2.955 (2)	106
C16—H16*A*⋯O5	0.93	2.51	2.896 (3)	105
C17—H17*C*⋯O3^ii^	0.96	2.46	3.361 (3)	156
C18—H18*A*⋯O4	0.97	2.46	2.799 (2)	100
C18—H18*B*⋯O5^i^	0.97	2.47	3.056 (2)	119
C20—H20*A*⋯O9	0.93	2.53	2.907 (2)	104
C24—H24*A*⋯O9^iii^	0.93	2.45	3.251 (2)	144
C25—H25*B*⋯O8^iv^	0.96	2.56	3.271 (3)	131
C26—H26*B*⋯O7	0.97	2.45	2.851 (2)	105
C29—H29*E*⋯O9^v^	0.96	2.55	3.504 (3)	172
C28—H28*D*⋯*Cg*1^vi^	0.96	3.25	3.888 (3)	125

Symmetry codes: (i) 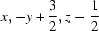; (ii) 
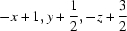
; (iii) 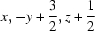; (iv) 
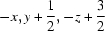
; (v) 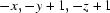; (vi) 
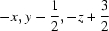
. *Cg*1 is the centroid of the C19–C24 ring.

**Fig. 1.**
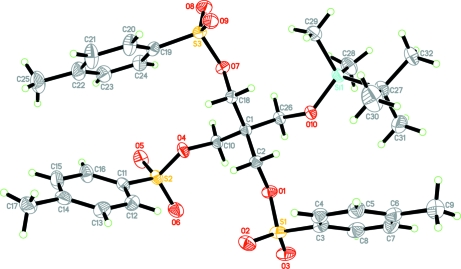
The asymmetric unit of (I), showing 50% probability displacement ellipsoids and the atomic numbering.

**Fig. 2.**
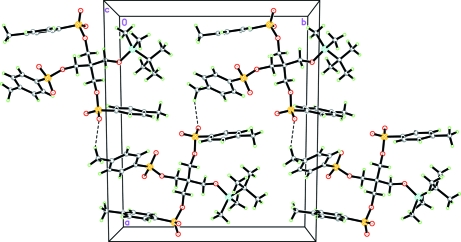
The crystal packing of (I), viewed down the c axis, showing screw chains running along the b axis. Hydrogen bonds are shown as dashed lines.

## supplementary crystallographic information

### Comment

Radio-immuno-imaging (RII) and radio-immuno-therapy (RIT) rely on the ability of a bifunctional molecule with a radioactive unit combined with a receptor-specific substrate such as 2-nitroimidazole unit which can act as a hypoxia marker (Abdel-Jalil *et al.*, 2006; Monge *et al.*, 2001; Nagasawa *et al.*, 2006). This approach allows radiopharmaceuticals to be delivered specifically to malignant tissue while minimizing the risk of unspecific irradiation of sane tissue. As part of our research on the synthesis of new potential hypoxia markers, we report herein the synthesis and crystal structure of the *O*-(*tert*-Butyl- dimethylsilyl)-tris[*O-*(4-methylphenylsulfonyl)]-pentaerythritol (I)

In the title molecule (Fig. 1), the configurations of the Si^V^ and central C atoms are tetrahedral. The interplanar angles between the three phenyl rings of the three 4-methylphenylsulfonyl group are 41.15 (10)°, 18.11 (10)° and 44.09 (10)° for *A*/*B*, *A*/*C* and *B*/*C*, respectively, where ring A is C3–C8, ring B is C11–C16 and ring C is C19–C24. Atoms O1, O7, C1, C2 and C18 lie on the same plane, with the most deviation of 0.011 (2) Å for atom C1. The dihedral angles between the O1/O7/C1/C2/C18 plane and the mean plane of phenyl rings *A*, *B* and *C* are 71.26 (12)°, 55.52 (12)° and 86.79 (13)°, respectively. The conformation of the three 4-methylphenylsulfonyl groups with respect to the pentaerythritol unit, (C1/C2/C10/C18/C26/O1/O4/O7/O10), can be indicated by the torsion angles S1–O1–C2–C1 = -172.71 (11)°, S2–O4–C10–C1 = -155.23 (12)° and S3–O7–C18–C1 = -168.07 (11)°.

The crystal packing of (I) in Fig. 2 shows that the molecules are linked into screw chains along the *b* axis by weak C17—H17C···O3 (symmetry code: 1 - *x*, 1/2 + *y*, 3/2 - *z*) and C25—H25B···O8 (symmetry code:-*x*, 1/2 + *y*, 3/2 - *z*) interactions (Table 1). The crystal is stabilized by weak C—H···O intra- and intermolecular interactions (Table 1) and further stabilized by C—H···π interactions (Table 1); *Cg*1 is the centroid of C19–C24 ring.

### Experimental

The title compound was synthesized by disolving the 2,2-hydroxymethyl-2-(*tert*-butyldimethylsilyloxymethyl)-propan-1,3-diol (1.05 g, 4.0 mmol) in dry pyridine (40 ml) and tosyl chloride (3.05 g, 16.0 mmol) was then added. The reaction mixture was stirred for 24 h at room temperature, after which it was poured into ice-water (250 ml) and extracted with CH_2_Cl_2_ (250 ml). The organic layer was washed with water (250 ml), dried with MgSO_4_ and concentrated. The solid residue was purified by recrystalization with warm ethanol to afford the title compound as a white solid 2.27 g (yield: 80%). Colourless single crystals of the title compound suitable for *x*-ray structure determination were recrystallized from ethanol by slow evaporation of the solvent at room temperature [m.p. 371 K].

### Refinement

All H atoms were placed in calculated positions, with C—H = 0.93 Å, *U*_iso_ = 1.2*U*_eq_(C) for aromatic, C—H = 0.97 Å, *U*_iso_ = 1.2*U*_eq_(C) for CH_2_ and C—H = 0.96 Å, *U*_iso_ = 1.5*U*_eq_(C) for CH_3_ atoms. A rotating group model was used for the methyl groups.

### Crystal data

**Table:**

C_32_H_44_O_10_S_3_Si	*F*_000_ = 1512
*M**_r_* = 712.94	*D*_x_ = 1.314 Mg m^−^^3^
Monoclinic, *P*2_1_/*c*	Melting point: 371 K
Hall symbol: -P 2ybc	Mo *K*α radiation λ = 0.71073 Å
*a* = 19.0551 (3) Å	Cell parameters from 10507 reflections
*b* = 16.4726 (3) Å	θ = 1.1–30.0º
*c* = 11.6751 (2) Å	µ = 0.29 mm^−^^1^
β = 100.4250 (10)º	*T* = 100.0 (1) K
*V* = 3604.17 (11) Å^3^	Block, colourless
*Z* = 4	0.51 × 0.22 × 0.21 mm

### Data collection

**Table:**

Bruker SMART APEX2 CCD area-detector diffractometer	10507 independent reflections
Radiation source: fine-focus sealed tube	7229 reflections with *I* > 2σ(*I*)
Monochromator: graphite	*R*_int_ = 0.061
Detector resolution: 8.33 pixels mm^-1^	θ_max_ = 30.0º
*T* = 100.0(1) K	θ_min_ = 1.1º
ω scans	*h* = −26→26
Absorption correction: multi-scan(SADABS; Bruker, 2005)	*k* = −20→23
*T*_min_ = 0.865, *T*_max_ = 0.941	*l* = −16→16
45850 measured reflections	

### Refinement

**Table:**

Refinement on *F*^2^	Secondary atom site location: difference Fourier map
Least-squares matrix: full	Hydrogen site location: inferred from neighbouring sites
*R*[*F*^2^ > 2σ(*F*^2^)] = 0.049	H-atom parameters constrained
*wR*(*F*^2^) = 0.128	*w* = 1/[σ^2^(*F*_o_^2^) + (0.0542*P*)^2^ + 0.5781*P*] where *P* = (*F*_o_^2^ + 2*F*_c_^2^)/3
*S* = 1.08	(Δ/σ)_max_ = 0.001
10507 reflections	Δρ_max_ = 0.57 e Å^−^^3^
423 parameters	Δρ_min_ = −0.37 e Å^−^^3^
Primary atom site location: structure-invariant direct methods	Extinction correction: none

### Special details

**Table:**

Experimental. The low-temparture data was collected with the Oxford Cryosystem Cobra low-temperature attachment.
Geometry. All e.s.d.'s (except the e.s.d. in the dihedral angle between two l.s. planes) are estimated using the full covariance matrix. The cell e.s.d.'s are taken into account individually in the estimation of e.s.d.'s in distances, angles and torsion angles; correlations between e.s.d.'s in cell parameters are only used when they are defined by crystal symmetry. An approximate (isotropic) treatment of cell e.s.d.'s is used for estimating e.s.d.'s involving l.s. planes.
Refinement. Refinement of *F*^2^ against ALL reflections. The weighted *R*-factor *wR* and goodness of fit *S* are based on *F*^2^, conventional *R*-factors *R* are based on *F*, with *F* set to zero for negative *F*^2^. The threshold expression of *F*^2^ > σ(*F*^2^) is used only for calculating *R*-factors(gt) *etc*. and is not relevant to the choice of reflections for refinement. *R*-factors based on *F*^2^ are statistically about twice as large as those based on *F*, and *R*- factors based on ALL data will be even larger.

### Fractional atomic coordinates and isotropic or equivalent isotropic displacement parameters (Å^2^)

**Table:**

	*x*	*y*	*z*	*U*_iso_*/*U*_eq_	
S1	0.43907 (2)	0.59668 (3)	0.80628 (4)	0.02743 (12)	
S2	0.29545 (3)	0.84432 (3)	0.88867 (4)	0.02536 (11)	
S3	0.04614 (2)	0.70517 (3)	0.62782 (4)	0.02418 (11)	
Si1	0.15861 (3)	0.41864 (3)	0.71109 (5)	0.02434 (12)	
O1	0.35890 (7)	0.60108 (8)	0.82612 (11)	0.0251 (3)	
O2	0.45358 (7)	0.66628 (9)	0.74133 (12)	0.0322 (3)	
O3	0.47849 (7)	0.58293 (10)	0.92048 (12)	0.0360 (4)	
O4	0.25506 (7)	0.77595 (7)	0.80747 (11)	0.0255 (3)	
O5	0.24793 (8)	0.87813 (9)	0.95759 (12)	0.0346 (3)	
O6	0.36172 (8)	0.81218 (9)	0.94769 (13)	0.0364 (4)	
O7	0.11116 (6)	0.66522 (8)	0.71203 (11)	0.0231 (3)	
O8	−0.01360 (7)	0.68777 (9)	0.68183 (13)	0.0350 (3)	
O9	0.04655 (7)	0.67929 (8)	0.51126 (12)	0.0306 (3)	
O10	0.22073 (7)	0.48853 (7)	0.75227 (11)	0.0237 (3)	
C1	0.23508 (9)	0.63386 (10)	0.77255 (15)	0.0201 (3)	
C2	0.30457 (9)	0.62322 (11)	0.72675 (15)	0.0218 (4)	
H2A	0.2995	0.5809	0.6681	0.026*	
H2B	0.3174	0.6734	0.6922	0.026*	
C3	0.44109 (10)	0.50976 (12)	0.72072 (17)	0.0271 (4)	
C4	0.42767 (10)	0.51678 (13)	0.60011 (17)	0.0292 (4)	
H4A	0.4179	0.5672	0.5650	0.035*	
C5	0.42907 (11)	0.44783 (13)	0.53309 (19)	0.0323 (5)	
H5A	0.4194	0.4523	0.4524	0.039*	
C6	0.44467 (10)	0.37191 (13)	0.5836 (2)	0.0342 (5)	
C7	0.45830 (11)	0.36682 (14)	0.7042 (2)	0.0385 (5)	
H7A	0.4691	0.3166	0.7393	0.046*	
C8	0.45632 (11)	0.43424 (13)	0.77347 (19)	0.0345 (5)	
H8A	0.4650	0.4295	0.8542	0.041*	
C9	0.44730 (13)	0.29802 (14)	0.5085 (2)	0.0472 (6)	
H9A	0.4627	0.2520	0.5571	0.071*	
H9B	0.4802	0.3073	0.4566	0.071*	
H9C	0.4007	0.2877	0.4640	0.071*	
C10	0.24235 (10)	0.69985 (10)	0.86520 (16)	0.0226 (4)	
H10A	0.1991	0.7036	0.8977	0.027*	
H10B	0.2819	0.6879	0.9277	0.027*	
C11	0.30845 (10)	0.91365 (11)	0.78179 (16)	0.0241 (4)	
C12	0.35159 (11)	0.89374 (13)	0.70172 (17)	0.0297 (4)	
H12A	0.3732	0.8430	0.7038	0.036*	
C13	0.36207 (11)	0.95052 (13)	0.61867 (18)	0.0312 (4)	
H13A	0.3904	0.9373	0.5644	0.037*	
C14	0.33077 (10)	1.02698 (12)	0.61554 (17)	0.0296 (4)	
C15	0.28933 (12)	1.04578 (13)	0.6978 (2)	0.0366 (5)	
H15A	0.2690	1.0971	0.6975	0.044*	
C16	0.27748 (12)	0.98965 (12)	0.78057 (19)	0.0335 (5)	
H16A	0.2490	1.0029	0.8347	0.040*	
C17	0.34224 (12)	1.08795 (14)	0.5246 (2)	0.0377 (5)	
H17A	0.3133	1.1350	0.5301	0.057*	
H17B	0.3291	1.0643	0.4487	0.057*	
H17C	0.3916	1.1035	0.5372	0.057*	
C18	0.17788 (9)	0.65611 (11)	0.66832 (15)	0.0205 (4)	
H18A	0.1901	0.7065	0.6336	0.025*	
H18B	0.1734	0.6137	0.6098	0.025*	
C19	0.06525 (10)	0.80900 (11)	0.63863 (17)	0.0249 (4)	
C20	0.06732 (12)	0.85348 (12)	0.53912 (18)	0.0352 (5)	
H20A	0.0617	0.8282	0.4668	0.042*	
C21	0.07791 (14)	0.93662 (13)	0.5490 (2)	0.0439 (6)	
H21A	0.0790	0.9672	0.4823	0.053*	
C22	0.08685 (12)	0.97525 (13)	0.6565 (2)	0.0373 (5)	
C23	0.08474 (12)	0.92879 (13)	0.75492 (19)	0.0352 (5)	
H23A	0.0910	0.9537	0.8275	0.042*	
C24	0.07355 (11)	0.84620 (12)	0.74651 (18)	0.0308 (4)	
H24A	0.0716	0.8157	0.8129	0.037*	
C25	0.09677 (14)	1.06606 (13)	0.6661 (2)	0.0502 (7)	
H25A	0.1296	1.0788	0.7363	0.075*	
H25B	0.0517	1.0915	0.6680	0.075*	
H25C	0.1154	1.0857	0.6002	0.075*	
C26	0.21518 (10)	0.55424 (10)	0.82791 (15)	0.0216 (4)	
H26A	0.2469	0.5456	0.9017	0.026*	
H26B	0.1668	0.5578	0.8426	0.026*	
C27	0.20588 (11)	0.33913 (12)	0.63907 (19)	0.0321 (5)	
C28	0.12636 (13)	0.37836 (14)	0.8409 (2)	0.0406 (5)	
H28D	0.0947	0.4171	0.8663	0.061*	
H28E	0.1663	0.3689	0.9024	0.061*	
H28F	0.1013	0.3283	0.8211	0.061*	
C29	0.08257 (12)	0.46319 (13)	0.6089 (2)	0.0418 (6)	
H29D	0.0614	0.5058	0.6474	0.063*	
H29E	0.0476	0.4219	0.5839	0.063*	
H29F	0.0993	0.4850	0.5425	0.063*	
C30	0.23327 (17)	0.37496 (18)	0.5347 (2)	0.0607 (8)	
H28A	0.2574	0.3336	0.4988	0.091*	
H28B	0.2658	0.4185	0.5605	0.091*	
H28C	0.1938	0.3954	0.4793	0.091*	
C31	0.26994 (13)	0.30612 (15)	0.7265 (3)	0.0537 (7)	
H29A	0.2944	0.2659	0.6890	0.081*	
H29B	0.2533	0.2821	0.7915	0.081*	
H29C	0.3020	0.3498	0.7534	0.081*	
C32	0.15490 (13)	0.26888 (14)	0.5985 (2)	0.0460 (6)	
H30A	0.1803	0.2264	0.5671	0.069*	
H30B	0.1166	0.2878	0.5395	0.069*	
H30C	0.1358	0.2483	0.6634	0.069*	

### Atomic displacement parameters (Å^2^)

**Table:**

	*U*^11^	*U*^22^	*U*^33^	*U*^12^	*U*^13^	*U*^23^
S1	0.0203 (2)	0.0361 (3)	0.0249 (2)	−0.00249 (19)	0.00168 (19)	0.0038 (2)
S2	0.0322 (3)	0.0219 (2)	0.0211 (2)	−0.00499 (18)	0.00224 (19)	−0.00392 (18)
S3	0.0212 (2)	0.0247 (2)	0.0258 (2)	0.00012 (17)	0.00182 (18)	0.00147 (18)
Si1	0.0269 (3)	0.0195 (2)	0.0257 (3)	−0.0037 (2)	0.0020 (2)	0.0008 (2)
O1	0.0210 (6)	0.0326 (7)	0.0207 (6)	0.0006 (5)	0.0015 (5)	0.0045 (6)
O2	0.0303 (7)	0.0351 (8)	0.0309 (7)	−0.0093 (6)	0.0048 (6)	0.0038 (6)
O3	0.0250 (7)	0.0525 (10)	0.0276 (7)	0.0001 (6)	−0.0030 (6)	0.0058 (7)
O4	0.0355 (7)	0.0178 (6)	0.0209 (6)	−0.0054 (5)	−0.0007 (6)	−0.0012 (5)
O5	0.0491 (9)	0.0306 (8)	0.0275 (7)	−0.0038 (7)	0.0163 (7)	−0.0046 (6)
O6	0.0366 (8)	0.0333 (8)	0.0342 (8)	−0.0071 (6)	−0.0071 (7)	0.0011 (7)
O7	0.0206 (6)	0.0270 (7)	0.0217 (6)	0.0029 (5)	0.0040 (5)	0.0027 (5)
O8	0.0236 (7)	0.0365 (8)	0.0461 (9)	−0.0015 (6)	0.0097 (6)	0.0051 (7)
O9	0.0334 (8)	0.0298 (7)	0.0254 (7)	0.0004 (6)	−0.0032 (6)	−0.0028 (6)
O10	0.0253 (7)	0.0189 (6)	0.0267 (7)	−0.0022 (5)	0.0041 (5)	−0.0032 (5)
C1	0.0223 (9)	0.0195 (8)	0.0180 (8)	−0.0011 (7)	0.0019 (7)	−0.0001 (7)
C2	0.0208 (9)	0.0237 (9)	0.0197 (9)	−0.0002 (7)	0.0003 (7)	0.0028 (7)
C3	0.0203 (9)	0.0332 (10)	0.0280 (10)	0.0024 (8)	0.0052 (8)	0.0054 (8)
C4	0.0251 (10)	0.0319 (10)	0.0311 (10)	0.0017 (8)	0.0064 (8)	0.0065 (9)
C5	0.0285 (10)	0.0345 (11)	0.0348 (11)	0.0018 (8)	0.0079 (9)	0.0015 (9)
C6	0.0219 (10)	0.0341 (11)	0.0476 (13)	0.0011 (8)	0.0087 (9)	0.0018 (10)
C7	0.0315 (11)	0.0320 (11)	0.0523 (14)	0.0056 (9)	0.0080 (10)	0.0128 (10)
C8	0.0285 (11)	0.0398 (12)	0.0342 (11)	0.0030 (9)	0.0034 (9)	0.0123 (9)
C9	0.0420 (13)	0.0352 (12)	0.0650 (17)	0.0000 (10)	0.0111 (12)	−0.0033 (12)
C10	0.0274 (9)	0.0193 (8)	0.0203 (9)	−0.0030 (7)	0.0023 (7)	0.0020 (7)
C11	0.0259 (9)	0.0223 (9)	0.0237 (9)	−0.0044 (7)	0.0033 (8)	−0.0037 (7)
C12	0.0295 (10)	0.0291 (10)	0.0305 (10)	0.0031 (8)	0.0057 (8)	−0.0007 (8)
C13	0.0293 (10)	0.0376 (11)	0.0281 (10)	0.0026 (9)	0.0089 (8)	0.0018 (9)
C14	0.0257 (10)	0.0321 (10)	0.0295 (10)	−0.0073 (8)	0.0007 (8)	0.0009 (9)
C15	0.0427 (12)	0.0224 (10)	0.0469 (13)	0.0005 (9)	0.0139 (10)	0.0020 (9)
C16	0.0431 (12)	0.0240 (10)	0.0377 (12)	−0.0004 (9)	0.0193 (10)	−0.0032 (9)
C17	0.0345 (12)	0.0372 (12)	0.0402 (12)	−0.0077 (9)	0.0039 (10)	0.0097 (10)
C18	0.0211 (9)	0.0200 (8)	0.0205 (8)	0.0004 (7)	0.0040 (7)	0.0002 (7)
C19	0.0245 (9)	0.0240 (9)	0.0268 (10)	0.0042 (7)	0.0066 (8)	0.0004 (8)
C20	0.0541 (14)	0.0270 (10)	0.0282 (11)	0.0088 (9)	0.0177 (10)	0.0017 (8)
C21	0.0711 (17)	0.0256 (11)	0.0428 (13)	0.0050 (11)	0.0312 (13)	0.0046 (10)
C22	0.0398 (12)	0.0273 (11)	0.0489 (13)	0.0036 (9)	0.0187 (11)	−0.0034 (10)
C23	0.0397 (12)	0.0329 (11)	0.0330 (11)	0.0020 (9)	0.0066 (10)	−0.0075 (9)
C24	0.0367 (11)	0.0299 (10)	0.0256 (10)	0.0012 (8)	0.0052 (9)	−0.0002 (8)
C25	0.0618 (16)	0.0251 (11)	0.0679 (18)	0.0025 (11)	0.0229 (14)	−0.0057 (11)
C26	0.0264 (9)	0.0196 (8)	0.0185 (8)	−0.0022 (7)	0.0029 (7)	0.0001 (7)
C27	0.0335 (11)	0.0269 (10)	0.0354 (11)	−0.0041 (8)	0.0046 (9)	−0.0093 (9)
C28	0.0474 (14)	0.0353 (12)	0.0417 (13)	−0.0123 (10)	0.0146 (11)	0.0011 (10)
C29	0.0366 (12)	0.0289 (11)	0.0534 (14)	−0.0051 (9)	−0.0094 (11)	0.0037 (10)
C30	0.081 (2)	0.0573 (17)	0.0529 (16)	−0.0102 (15)	0.0374 (15)	−0.0153 (14)
C31	0.0426 (14)	0.0424 (14)	0.0708 (18)	0.0112 (11)	−0.0043 (13)	−0.0224 (13)
C32	0.0474 (14)	0.0300 (12)	0.0576 (15)	−0.0050 (10)	0.0016 (12)	−0.0173 (11)

### Geometric parameters (Å, °)

**Table:**

S1—O3	1.4250 (14)	C13—C14	1.391 (3)
S1—O2	1.4289 (14)	C13—H13A	0.9300
S1—O1	1.5875 (13)	C14—C15	1.385 (3)
S1—C3	1.750 (2)	C14—C17	1.505 (3)
S2—O6	1.4265 (15)	C15—C16	1.385 (3)
S2—O5	1.4288 (14)	C15—H15A	0.9300
S2—O4	1.5797 (13)	C16—H16A	0.9300
S2—C11	1.7420 (19)	C17—H17A	0.9600
S3—O8	1.4266 (14)	C17—H17B	0.9600
S3—O9	1.4274 (14)	C17—H17C	0.9600
S3—O7	1.5791 (13)	C18—H18A	0.9700
S3—C19	1.7484 (19)	C18—H18B	0.9700
Si1—O10	1.6588 (13)	C19—C20	1.380 (3)
Si1—C29	1.854 (2)	C19—C24	1.384 (3)
Si1—C28	1.858 (2)	C20—C21	1.386 (3)
Si1—C27	1.873 (2)	C20—H20A	0.9300
O1—C2	1.455 (2)	C21—C22	1.390 (3)
O4—C10	1.464 (2)	C21—H21A	0.9300
O7—C18	1.461 (2)	C22—C23	1.387 (3)
O10—C26	1.413 (2)	C22—C25	1.509 (3)
C1—C10	1.522 (2)	C23—C24	1.378 (3)
C1—C18	1.524 (2)	C23—H23A	0.9300
C1—C2	1.525 (2)	C24—H24A	0.9300
C1—C26	1.540 (2)	C25—H25A	0.9600
C2—H2A	0.9700	C25—H25B	0.9600
C2—H2B	0.9700	C25—H25C	0.9600
C3—C4	1.390 (3)	C26—H26A	0.9700
C3—C8	1.395 (3)	C26—H26B	0.9700
C4—C5	1.382 (3)	C27—C30	1.528 (3)
C4—H4A	0.9300	C27—C32	1.530 (3)
C5—C6	1.392 (3)	C27—C31	1.542 (3)
C5—H5A	0.9300	C28—H28D	0.9600
C6—C7	1.388 (3)	C28—H28E	0.9600
C6—C9	1.506 (3)	C28—H28F	0.9600
C7—C8	1.378 (3)	C29—H29D	0.9600
C7—H7A	0.9300	C29—H29E	0.9600
C8—H8A	0.9300	C29—H29F	0.9600
C9—H9A	0.9600	C30—H28A	0.9600
C9—H9B	0.9600	C30—H28B	0.9600
C9—H9C	0.9600	C30—H28C	0.9600
C10—H10A	0.9700	C31—H29A	0.9600
C10—H10B	0.9700	C31—H29B	0.9600
C11—C16	1.383 (3)	C31—H29C	0.9600
C11—C12	1.391 (3)	C32—H30A	0.9600
C12—C13	1.387 (3)	C32—H30B	0.9600
C12—H12A	0.9300	C32—H30C	0.9600
			
O3—S1—O2	120.27 (9)	C14—C15—H15A	119.3
O3—S1—O1	103.23 (8)	C16—C15—H15A	119.3
O2—S1—O1	108.94 (8)	C11—C16—C15	119.24 (19)
O3—S1—C3	109.98 (9)	C11—C16—H16A	120.4
O2—S1—C3	109.09 (9)	C15—C16—H16A	120.4
O1—S1—C3	104.00 (8)	C14—C17—H17A	109.5
O6—S2—O5	117.94 (9)	C14—C17—H17B	109.5
O6—S2—O4	108.54 (8)	H17A—C17—H17B	109.5
O5—S2—O4	109.04 (8)	C14—C17—H17C	109.5
O6—S2—C11	111.17 (9)	H17A—C17—H17C	109.5
O5—S2—C11	109.55 (9)	H17B—C17—H17C	109.5
O4—S2—C11	98.89 (8)	O7—C18—C1	106.71 (14)
O8—S3—O9	120.25 (9)	O7—C18—H18A	110.4
O8—S3—O7	103.88 (8)	C1—C18—H18A	110.4
O9—S3—O7	109.27 (8)	O7—C18—H18B	110.4
O8—S3—C19	109.69 (9)	C1—C18—H18B	110.4
O9—S3—C19	108.91 (9)	H18A—C18—H18B	108.6
O7—S3—C19	103.48 (8)	C20—C19—C24	121.00 (19)
O10—Si1—C29	110.21 (9)	C20—C19—S3	119.73 (15)
O10—Si1—C28	109.60 (9)	C24—C19—S3	119.14 (15)
C29—Si1—C28	109.34 (12)	C19—C20—C21	118.7 (2)
O10—Si1—C27	103.86 (8)	C19—C20—H20A	120.6
C29—Si1—C27	111.79 (11)	C21—C20—H20A	120.6
C28—Si1—C27	111.93 (10)	C20—C21—C22	121.3 (2)
C2—O1—S1	117.37 (11)	C20—C21—H21A	119.4
C10—O4—S2	115.94 (11)	C22—C21—H21A	119.4
C18—O7—S3	117.36 (11)	C23—C22—C21	118.6 (2)
C26—O10—Si1	125.64 (11)	C23—C22—C25	120.5 (2)
C10—C1—C18	110.83 (14)	C21—C22—C25	120.9 (2)
C10—C1—C2	110.97 (15)	C24—C23—C22	120.9 (2)
C18—C1—C2	106.77 (14)	C24—C23—H23A	119.6
C10—C1—C26	107.92 (14)	C22—C23—H23A	119.6
C18—C1—C26	110.20 (14)	C23—C24—C19	119.5 (2)
C2—C1—C26	110.16 (14)	C23—C24—H24A	120.2
O1—C2—C1	106.68 (13)	C19—C24—H24A	120.2
O1—C2—H2A	110.4	C22—C25—H25A	109.5
C1—C2—H2A	110.4	C22—C25—H25B	109.5
O1—C2—H2B	110.4	H25A—C25—H25B	109.5
C1—C2—H2B	110.4	C22—C25—H25C	109.5
H2A—C2—H2B	108.6	H25A—C25—H25C	109.5
C4—C3—C8	120.46 (19)	H25B—C25—H25C	109.5
C4—C3—S1	119.42 (15)	O10—C26—C1	109.77 (14)
C8—C3—S1	120.12 (16)	O10—C26—H26A	109.7
C5—C4—C3	119.10 (19)	C1—C26—H26A	109.7
C5—C4—H4A	120.5	O10—C26—H26B	109.7
C3—C4—H4A	120.5	C1—C26—H26B	109.7
C4—C5—C6	121.6 (2)	H26A—C26—H26B	108.2
C4—C5—H5A	119.2	C30—C27—C32	109.5 (2)
C6—C5—H5A	119.2	C30—C27—C31	108.7 (2)
C7—C6—C5	118.1 (2)	C32—C27—C31	108.70 (19)
C7—C6—C9	121.5 (2)	C30—C27—Si1	110.44 (16)
C5—C6—C9	120.5 (2)	C32—C27—Si1	109.76 (15)
C8—C7—C6	121.8 (2)	C31—C27—Si1	109.69 (15)
C8—C7—H7A	119.1	Si1—C28—H28D	109.5
C6—C7—H7A	119.1	Si1—C28—H28E	109.5
C7—C8—C3	119.0 (2)	H28D—C28—H28E	109.5
C7—C8—H8A	120.5	Si1—C28—H28F	109.5
C3—C8—H8A	120.5	H28D—C28—H28F	109.5
C6—C9—H9A	109.5	H28E—C28—H28F	109.5
C6—C9—H9B	109.5	Si1—C29—H29D	109.5
H9A—C9—H9B	109.5	Si1—C29—H29E	109.5
C6—C9—H9C	109.5	H29D—C29—H29E	109.5
H9A—C9—H9C	109.5	Si1—C29—H29F	109.5
H9B—C9—H9C	109.5	H29D—C29—H29F	109.5
O4—C10—C1	106.60 (14)	H29E—C29—H29F	109.5
O4—C10—H10A	110.4	C27—C30—H28A	109.5
C1—C10—H10A	110.4	C27—C30—H28B	109.5
O4—C10—H10B	110.4	H28A—C30—H28B	109.5
C1—C10—H10B	110.4	C27—C30—H28C	109.5
H10A—C10—H10B	108.6	H28A—C30—H28C	109.5
C16—C11—C12	120.62 (18)	H28B—C30—H28C	109.5
C16—C11—S2	119.02 (15)	C27—C31—H29A	109.5
C12—C11—S2	120.32 (15)	C27—C31—H29B	109.5
C13—C12—C11	119.21 (19)	H29A—C31—H29B	109.5
C13—C12—H12A	120.4	C27—C31—H29C	109.5
C11—C12—H12A	120.4	H29A—C31—H29C	109.5
C12—C13—C14	120.89 (19)	H29B—C31—H29C	109.5
C12—C13—H13A	119.6	C27—C32—H30A	109.5
C14—C13—H13A	119.6	C27—C32—H30B	109.5
C15—C14—C13	118.68 (19)	H30A—C32—H30B	109.5
C15—C14—C17	120.91 (19)	C27—C32—H30C	109.5
C13—C14—C17	120.41 (19)	H30A—C32—H30C	109.5
C14—C15—C16	121.34 (19)	H30B—C32—H30C	109.5
			
O3—S1—O1—C2	173.78 (13)	S2—C11—C12—C13	−178.92 (15)
O2—S1—O1—C2	44.86 (14)	C11—C12—C13—C14	0.8 (3)
C3—S1—O1—C2	−71.37 (14)	C12—C13—C14—C15	0.6 (3)
O6—S2—O4—C10	56.27 (14)	C12—C13—C14—C17	−179.71 (19)
O5—S2—O4—C10	−73.41 (14)	C13—C14—C15—C16	−1.4 (3)
C11—S2—O4—C10	172.24 (13)	C17—C14—C15—C16	178.9 (2)
O8—S3—O7—C18	166.39 (12)	C12—C11—C16—C15	0.6 (3)
O9—S3—O7—C18	36.88 (14)	S2—C11—C16—C15	178.17 (16)
C19—S3—O7—C18	−79.02 (13)	C14—C15—C16—C11	0.8 (3)
C29—Si1—O10—C26	71.63 (16)	S3—O7—C18—C1	168.07 (11)
C28—Si1—O10—C26	−48.75 (16)	C10—C1—C18—O7	−59.77 (18)
C27—Si1—O10—C26	−168.49 (14)	C2—C1—C18—O7	179.26 (13)
S1—O1—C2—C1	−172.71 (11)	C26—C1—C18—O7	59.62 (17)
C10—C1—C2—O1	59.75 (18)	O8—S3—C19—C20	−124.09 (17)
C18—C1—C2—O1	−179.37 (13)	O9—S3—C19—C20	9.39 (19)
C26—C1—C2—O1	−59.71 (18)	O7—S3—C19—C20	125.55 (17)
O3—S1—C3—C4	−158.68 (15)	O8—S3—C19—C24	51.81 (18)
O2—S1—C3—C4	−24.77 (18)	O9—S3—C19—C24	−174.70 (15)
O1—S1—C3—C4	91.34 (16)	O7—S3—C19—C24	−58.54 (17)
O3—S1—C3—C8	21.08 (19)	C24—C19—C20—C21	0.0 (3)
O2—S1—C3—C8	154.99 (15)	S3—C19—C20—C21	175.80 (18)
O1—S1—C3—C8	−88.89 (17)	C19—C20—C21—C22	0.4 (4)
C8—C3—C4—C5	0.5 (3)	C20—C21—C22—C23	−0.2 (4)
S1—C3—C4—C5	−179.74 (15)	C20—C21—C22—C25	−178.7 (2)
C3—C4—C5—C6	−1.0 (3)	C21—C22—C23—C24	−0.4 (3)
C4—C5—C6—C7	0.6 (3)	C25—C22—C23—C24	178.0 (2)
C4—C5—C6—C9	−178.76 (19)	C22—C23—C24—C19	0.9 (3)
C5—C6—C7—C8	0.3 (3)	C20—C19—C24—C23	−0.6 (3)
C9—C6—C7—C8	179.7 (2)	S3—C19—C24—C23	−176.47 (16)
C6—C7—C8—C3	−0.8 (3)	Si1—O10—C26—C1	−129.79 (13)
C4—C3—C8—C7	0.4 (3)	C10—C1—C26—O10	−169.26 (14)
S1—C3—C8—C7	−179.36 (16)	C18—C1—C26—O10	69.59 (18)
S2—O4—C10—C1	−155.23 (12)	C2—C1—C26—O10	−47.96 (19)
C18—C1—C10—O4	−54.42 (18)	O10—Si1—C27—C30	−60.39 (18)
C2—C1—C10—O4	64.04 (18)	C29—Si1—C27—C30	58.41 (19)
C26—C1—C10—O4	−175.17 (14)	C28—Si1—C27—C30	−178.54 (17)
O6—S2—C11—C16	−128.23 (17)	O10—Si1—C27—C32	178.73 (15)
O5—S2—C11—C16	3.90 (19)	C29—Si1—C27—C32	−62.47 (19)
O4—S2—C11—C16	117.85 (17)	C28—Si1—C27—C32	60.58 (19)
O6—S2—C11—C12	49.36 (18)	O10—Si1—C27—C31	59.37 (17)
O5—S2—C11—C12	−178.51 (15)	C29—Si1—C27—C31	178.17 (16)
O4—S2—C11—C12	−64.57 (17)	C28—Si1—C27—C31	−58.78 (19)
C16—C11—C12—C13	−1.4 (3)		

### Hydrogen-bond geometry (Å, °)

**Table:**

*D*—H···*A*	*D*—H	H···*A*	*D*···*A*	*D*—H···*A*
C2—H2A···O5^i^	0.97	2.57	3.133 (2)	117
C2—H2B···O2	0.97	2.56	2.902 (2)	101
C4—H4A···O6^i^	0.93	2.54	3.446 (3)	166
C10—H10B···O1	0.97	2.50	2.856 (2)	102
C10—H10B···O6	0.97	2.54	2.955 (2)	106
C16—H16A···O5	0.93	2.51	2.896 (3)	105
C17—H17C···O3^ii^	0.96	2.46	3.361 (3)	156
C18—H18A···O4	0.97	2.46	2.799 (2)	100
C18—H18B···O5^i^	0.97	2.47	3.056 (2)	119
C20—H20A···O9	0.93	2.54	2.907 (2)	104
C24—H24A···O9^iii^	0.93	2.45	3.251 (2)	144
C25—H25B···O8^iv^	0.96	2.57	3.271 (3)	131
C26—H26B···O7	0.97	2.45	2.851 (2)	105
C29—H29E···O9^v^	0.96	2.55	3.504 (3)	172
C28—H28D···Cg1^vi^	0.96	3.25	3.888 (3)	125

Symmetry codes: (i) *x*, −*y*+3/2, *z*−1/2;  (ii) −*x*+1, *y*+1/2, −*z*+3/2; (iii) *x*, −*y*+3/2, *z*+1/2; (iv) −*x*, *y*+1/2, −*z*+3/2; (v) −*x*, −*y*+1, −*z*+1; (vi) −*x*, *y*−1/2, −*z*+3/2.
